# Robust trajectory tracking control of an underwater robotic manipulator using an IPSO-PID framework under hydrodynamic and ocean-current disturbances

**DOI:** 10.3389/frobt.2026.1915434

**Published:** 2026-08-28

**Authors:** Jinming Yao, Jiahang Yang, Chunxu Li

**Affiliations:** 1 School of Rehabilitation Sciences and Engineering, University of Health and Rehabilitation Sciences, Qingdao, China; 2 College of Mechanical and Electrical Engineering, Hohai University, Changzhou, China

**Keywords:** hydrodynamic disturbance, IPSO-PID, MATLAB/Simulink, ocean-current disturbance, robust control, trajectory tracking control, underwater robotic manipulator

## Abstract

Robust trajectory tracking is a fundamental requirement for underwater robotic manipulators operating in disturbed marine environments. However, hydrodynamic damping, added mass, buoyancy effects, multi-joint coupling, and external ocean-current disturbances can significantly degrade joint tracking accuracy and control stability. To address this problem, this paper proposes an improved particle swarm optimization-based PID (IPSO-PID) control framework for robust trajectory tracking of a 6-DOF underwater manipulator. A UR5-type underwater manipulator is modeled using the D-H parameter method, while its dynamic behavior is formulated based on the Lagrange method. To represent underwater environmental effects, the Morison equation is introduced to describe fluid drag and added-mass forces, and a compound ocean-current disturbance model consisting of a constant bias load and a low-frequency periodic component is further constructed. In the control framework, the standard PSO algorithm is improved by incorporating a linearly decreasing inertia weight and adaptive learning factors, thereby enhancing global exploration in the early optimization stage and local convergence in the later stage. A comprehensive fitness function is designed by jointly considering trajectory tracking error, error convergence behavior, and control effort, enabling coordinated optimization of the PID parameters for all six joints. Comparative simulations are conducted in MATLAB/Simulink using conventional PID, fuzzy PID, standard PSO-PID, and the proposed IPSO-PID controller. The results show that the proposed controller achieves better tracking accuracy and stronger disturbance rejection capability than the comparison methods. Under compound ocean-current disturbance, the overall mean absolute error across all six joints is reduced from approximately 0.0044 rad with the conventional PID controller to approximately 0.0014 rad with the IPSO-PID controller, corresponding to a reduction of about 68.2%. These results indicate that the proposed control framework can effectively improve the stability and robustness of underwater manipulator trajectory tracking, providing a feasible solution for underwater robotic tasks such as inspection, grasping, maintenance, and marine intervention.

## 1 Introduction

Underwater manipulators are key execution devices for underwater robots in marine exploration, target grasping, equipment maintenance, and deep-sea operations. Compared with terrestrial manipulators, underwater manipulators operate in more complex environments, where their motion is affected by hydrodynamic factors such as added mass, fluid resistance, viscous damping, buoyancy, and ocean-current disturbances. These factors increase the nonlinearity and time-varying characteristics of the system, leading to joint response fluctuations, increased end-effector trajectory deviations, and reduced control stability. In practical underwater operations, such tracking deviations may cause inaccurate target positioning, unstable contact during grasping or maintenance, and unintended collisions with surrounding structures, thereby increasing operational risk and reducing task reliability. Therefore, high-precision trajectory tracking is essential not only for task accuracy but also for ensuring the safety and stability of underwater operations under complex hydrodynamic conditions (Chang et al., 2020; McLain and Rock, 1998; Kolodziejczyk, 2018).

For the trajectory tracking control of underwater manipulators, conventional PID control is still widely used in robotic joint control due to its simple structure and ease of implementation. However, the parameters of conventional PID controllers usually depend on manual empirical tuning. When the manipulator is affected by hydrodynamic disturbances and multi-joint coupling, it is difficult to simultaneously ensure response speed, steady-state accuracy, and disturbance rejection capability. Fuzzy PID control can adjust control parameters online according to the tracking error and its rate of change, thereby improving the adaptability of the control system to some extent. Nevertheless, its initial parameters, membership functions, and fuzzy rules still rely on empirical design. In recent years, particle swarm optimization has been introduced into control parameter tuning to improve the search efficiency of PID parameters and the control performance. However, standard PSO is prone to reduced population diversity, premature convergence, and insufficient local search capability in high-dimensional multi-parameter optimization, which limits its optimization performance in trajectory tracking control of multi-joint underwater manipulators (Minorsky, 1922; Kennedy and Eberhart, 1995; Tao et al., 2021).

To address these challenges, this paper develops an IPSO-PID joint-space control framework for robust trajectory tracking of a 6-DOF underwater robotic manipulator under hydrodynamic and ocean-current disturbances. A UR5-type underwater manipulator is used as the research object, and its kinematic model is established using the D-H parameter method. Based on the Lagrange formulation and the Morison equation, a dynamic model incorporating rigid-body dynamics, buoyancy, hydrodynamic drag, and added-mass effects is constructed. In addition, a compound ocean-current disturbance model, consisting of a constant bias load and a low-frequency periodic component, is introduced to represent persistent and time-varying external disturbances in underwater operations. For the control design, an improved PSO algorithm with a linearly decreasing inertia weight and adaptive learning factors is developed to optimize the PID parameters of all six joints in a coordinated manner. A comprehensive fitness function is further designed by considering trajectory tracking error, error convergence behavior, and control effort. Comparative simulations are conducted in MATLAB/Simulink under both nominal hydrodynamic conditions and compound ocean-current disturbances. The proposed method is compared with conventional PID, fuzzy PID, and standard PSO-PID controllers to evaluate its tracking accuracy, convergence performance, and disturbance rejection capability.

The main contributions of this paper are summarized as follows:

1. A trajectory tracking simulation model of a 6-DOF underwater manipulator considering rigid-body dynamics and Morison hydrodynamic terms is established, providing a basis for control algorithm validation under hydrodynamic disturbance conditions.
2. An IPSO-PID control method is proposed. By introducing adaptive learning factors and a dynamic inertia weight, the global search capability and local optimization capability of the algorithm in the PID parameter space are enhanced, thereby improving the parameter optimization performance.
3. A comprehensive fitness function is designed for multi-joint trajectory tracking control, in which tracking accuracy, error convergence behavior, and control effort are jointly considered. This formulation enables the PID parameters of all six joints to be optimized in a coordinated manner rather than through independent manual tuning.
4. Comparative simulations are carried out under nominal hydrodynamic conditions and compound ocean-current disturbances. The results demonstrate that the proposed IPSO-PID controller achieves higher trajectory tracking accuracy and stronger disturbance rejection performance than conventional PID, fuzzy PID, and standard PSO-PID controllers.

## 2 Related work

### 2.1 Dynamics and hydrodynamic modeling of underwater manipulators

Compared with land-based manipulators, underwater manipulators are subject to additional hydrodynamic effects during motion, including added mass, viscous damping, drag, and buoyancy. These effects introduce stronger nonlinearities and time-varying characteristics into the system. Therefore, an appropriate hydrodynamic model is essential for improving the trajectory tracking performance of underwater manipulators. McLain and Rock established and experimentally validated a hydrodynamic model for an underwater manipulator, demonstrating that fluid-induced forces have a significant influence on the dynamic response of the system (McLain and Rock, 1998). Kolodziejczyk investigated the identification of transient hydrodynamic coefficients for a single-DOF underwater manipulator, providing a reference for hydrodynamic parameter modeling (Kolodziejczyk, 2018). Gupta and Pratiher developed a dynamic model for a two-link flexible underwater manipulator considering hydrodynamic effects, and validated the model experimentally using cascade PID control (Gupta and Pratiher, 2025). Duan et al. further analyzed the hydrodynamic coefficients of underwater manipulators under different configurations, showing that variations in manipulator posture can affect hydrodynamic parameters (Duan et al., 2024). Existing studies indicate that hydrodynamic modeling has a direct influence on the control performance of underwater manipulators (Zheng et al., 2023). However, for multi-DOF manipulators operating under complex motion conditions, a trade-off is still required between model accuracy and control implementability. Therefore, this paper adopts the relatively compact Morison equation to describe the dominant hydrodynamic effects and incorporates it into the trajectory tracking control simulation of the underwater manipulator.

### 2.2 Trajectory tracking control methods for underwater manipulators

The objective of trajectory tracking control for underwater manipulators is to enable the joints or end-effector to accurately follow the desired trajectory under hydrodynamic disturbances and model uncertainties. Traditional PID control remains a commonly used method in manipulator joint control due to its simple structure, ease of implementation, and relatively good stability (Minorsky, 1922). However, in underwater environments, manipulator systems are affected by multi-joint coupling, nonlinear hydrodynamic forces, and external disturbances. Therefore, it is usually difficult to obtain satisfactory dynamic response and steady-state accuracy by relying only on manually tuned PID parameters. To improve control performance, existing studies have adopted various methods to address underwater manipulator control problems, including genetic algorithm-tuned adaptive neural network control, inverse dynamics disturbance compensation, robust nonlinear model predictive control, fuzzy RBF neural networks, adaptive sliding mode control, and deep reinforcement learning (Salloom et al., 2020; Filaretov et al., 2021; Nikou et al., 2021; Yuan et al., 2019; Li et al., 2023; Gao et al., 2022). These methods can improve control accuracy and robustness to a certain extent. However, some of them suffer from complex structures, high computational cost, or difficult parameter design (Xue et al., 2022; Xiong et al., 2023; Trekel et al., 2025). Recent studies have further investigated adaptive fuzzy sliding-mode control, disturbance-observer-based adaptive sliding-mode control, and tube model predictive control for underwater manipulators and underwater vehicle–manipulator systems under hydrodynamic uncertainties and external disturbances (Zhang et al., 2024; Zhu et al., 2024; Xu et al., 2025). Therefore, introducing intelligent optimization algorithms into traditional PID control to achieve automatic parameter optimization provides an effective approach that balances implementation simplicity and control performance.

### 2.3 Application of intelligent optimization algorithms in PID parameter optimization

PID parameter optimization is an important factor affecting the trajectory tracking performance of manipulators (Zhang et al., 2023; Zhou et al., 2024). Traditional manual tuning is inefficient and makes it difficult to obtain globally optimal parameters under multi-joint coupling and hydrodynamic disturbances. Therefore, swarm intelligence algorithms, such as particle swarm optimization, genetic algorithms, grey wolf optimization, and ant lion optimization, have been widely used for controller parameter optimization (Kennedy and Eberhart, 1995; Faris et al., 2018). Among these methods, PSO is particularly suitable for the present study because it retains the simple structure of PID control while enabling the simultaneous optimization of multiple controller parameters (Kennedy and Eberhart, 1995). However, for the coordinated optimization of the 18 PID parameters of a 6-DOF underwater manipulator, standard PSO may suffer from premature particle aggregation, reduced population diversity, and insufficient local search capability in the high-dimensional parameter space (Tao et al., 2021). These limitations may lead to suboptimal controller gains and restrict trajectory tracking performance under multi-joint coupling and hydrodynamic disturbances. Therefore, rather than adopting a structurally more complex nonlinear controller, this study improves the PSO search mechanism by introducing a linearly decreasing inertia weight and adaptive learning factors. This choice preserves the implementation simplicity of PID control while enhancing global exploration in the early optimization stage and local convergence accuracy in the later stage.

## 3 Modeling of the underwater manipulator

### 3.1 Kinematic model of the 6-DOF underwater manipulator

To describe the geometric mapping relationship between the end-effector of the underwater manipulator and the joint variables, this paper takes a UR5-type 6-DOF serial manipulator as the research object and establishes its kinematic model using the D–H parameter method. The manipulator consists of six revolute joints connected in series, and its joint variables can be expressed as:

$$q={\left[q_{1},q_{2},q_{3},q_{4},q_{5},q_{6}\right]}^{T}$$

where $q_{i}$ denotes the rotation angle of the i -th joint. By establishing a local coordinate frame on each link, the pose transformation relationship from the base to the end-effector can be described using the homogeneous transformation matrix between adjacent links. According to the standard D–H modeling method, the homogeneous transformation matrix from frame i−1 to frame i can be expressed as:

$${i-1}_{T_{i}}=\left[\begin{array}{cccc} \cos q_{i} & -\sin q_{i}\cos\alpha_{i} & \sin q_{i}\sin\alpha_{i} & a_{i}\cos q_{i}\\ \sin q_{i} & \cos q_{i}\cos\alpha_{i} & -\cos q_{i}\sin\alpha_{i} & a_{i}\sin q_{i}\\ 0 & \sin\alpha_{i} & \cos\alpha_{i} & d_{i}\\ 0 & 0 & 0 & 1 \end{array}\right]$$

where $a_{i}$ is the link length, $\alpha_{i}$ is the link twist angle, $d_{i}$ is the link offset, and $q_{i}$ is the joint angle. According to the structural parameters of the UR5-type 6-DOF manipulator, its D–H parameters are listed in Table 1.

**TABLE 1:** D-H parameters of the UR5.

n	$a_{i}$	$\alpha_{i}$	$d_{i}$	$q_{i}$
1	0.0	π/2	0.089159	$q_{1}$
2	−0.425	0	0.0	$q_{2}$
3	−0.39225	0	0.0	$q_{3}$
4	0.0	π/2	0.10915	$q_{4}$
5	0.0	-π/2	0.09465	$q_{5}$
6	0.0	0	0.0823	$q_{6}$

**FIGURE 1:**
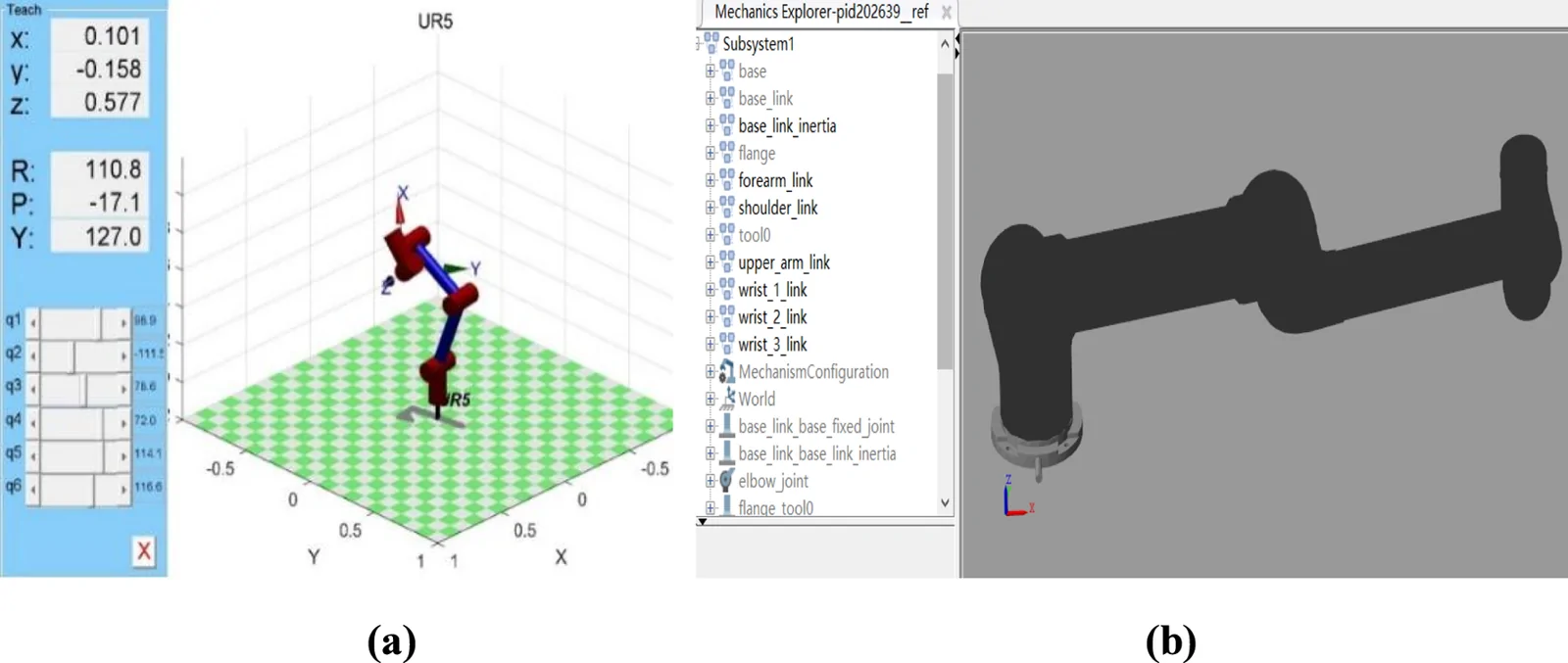
UR5-type 6-DOF manipulator models used in the simulation: **(a)** MATLAB Robotics Toolbox model; **(b)** Simscape Multibody model.

Based on the D–H parameters listed in Table 1, the UR5-type manipulator model was constructed using MATLAB Robotics Toolbox, as shown in Figure 1.

Based on the above D–H parameters, the pose of the end-effector relative to the base coordinate frame can be obtained by successively multiplying the homogeneous transformation matrices of each joint:

T60=T10T21T32T43T54T65

where ^0^ *T*_6_ is the homogeneous transformation matrix of the end-effector relative to the base coordinate frame, which can be further expressed as:

T6=0R60p6001×31=nxoxaxpxnyoyaypynzozazpz0001

where $R_{6}^{0}\in R^{3\times 3}$ represents the orientation matrix of the end-effector relative to the base coordinate frame, and $p_{6}^{0}={\left[p_{x},p_{y},p_{z}\right]}^{T}$ represents the position vector of the end-effector. Using a forward kinematic model, the position and orientation of the robotic arm’s end-effector in the base coordinate system are calculated based on the joint angles $q_{i}$ .

### 3.2 Dynamic model of the underwater manipulator

To achieve high-precision trajectory tracking control of the underwater manipulator, it is necessary to establish a dynamic model that can describe the relationship between the joint driving torques and the system motion states. Unlike manipulators operating in air, underwater manipulators are significantly affected by the surrounding fluid medium during motion, including added mass, viscous damping, fluid resistance, and buoyancy. Therefore, a conventional rigid-body dynamic model alone is insufficient to accurately describe the underwater motion characteristics. In this paper, the dynamic model of the manipulator is established based on the Lagrange method, and the hydrodynamic effects are incorporated into the system energy function and generalized torque terms.

For the rigid-body manipulator system, the Lagrange equation can be expressed as:

$$\frac{d}{dt}\left(\frac{\partial L}{\partial {\dot{q}}_{i}}\right)-\frac{\partial L}{\partial q_{i}}=\tau_{i}$$

where L is the Lagrangian function, $q_{i}$ and ${\dot{q}}_{i}$ denote the angular displacement and angular velocity of the i -th joint, respectively, and $\tau_{i}$ is the corresponding joint driving torque.

The Lagrangian function is defined as the difference between the rigid-body kinetic energy and the equivalent potential energy:

$$L\left(q,\dot{q}\right)=T\left(q,\dot{q}\right)-U\left(q\right)$$

where $T\left(q,\dot{q}\right)$ is the rigid-body kinetic energy of the manipulator and $U\left(q\right)$ is the equivalent potential energy considering the combined effects of gravity and buoyancy. The rigid-body kinetic energy can be expressed as:

$$T\left(q,\dot{q}\right)=\frac{1}{2}{\dot{q}}^{T}M_{r}\left(q\right)\dot{q}$$

where $M_{r}\left(q\right)$ denotes the rigid-body inertia matrix of the manipulator.

In the underwater environment, the potential energy of the manipulator is related not only to gravity but also to buoyancy. Therefore, the system potential energy can be expressed as the difference between gravitational potential energy and buoyancy potential energy:

$$U\left(q\right)=U_{g}\left(q\right)-U_{b}\left(q\right)$$

where $U_{g}\left(q\right)$ is the gravitational potential energy, and $U_{b}\left(q\right)$ is the buoyancy potential energy. Accordingly, the equivalent generalized force term under the combined action of gravity and buoyancy can be obtained as:

$$G_{b}\left(q\right)=\frac{\partial U\left(q\right)}{\partial q}$$

By substituting the above kinetic and potential energy terms into the Lagrange equation, the rigid-body dynamic model of the manipulator can be obtained as:

$$M_{r}\left(q\right)\ddot{q}+C_{r}\left(q,\dot{q}\right)\dot{q}+G_{b}\left(q\right)=\tau$$

where $M_{r}\left(q\right)$ is the rigid-body inertia matrix, $C_{r}\left(q,\dot{q}\right)\dot{q}$ represents the Coriolis and centrifugal torque generated by the rigid-body motion of the manipulator, $G_{b}\left(q\right)$ is the equivalent gravity–buoyancy term, and τ is the joint driving torque vector.

This equation describes the rigid-body subsystem used in the simulation. The hydrodynamic drag and added-mass effects are calculated separately by the underwater-environment module described in Section 3.3 and are introduced as external joint torques.

### 3.3 Hydrodynamic modeling based on the Morison equation

The fluid forces acting on an underwater manipulator during motion mainly include two components: the drag effect caused by the relative velocity between the links and the surrounding fluid, and the added-mass effect caused by the acceleration of the surrounding fluid driven by the accelerating links. Since the transverse dimensions of the manipulator links are much smaller than their lengths, the links can be reasonably approximated as slender circular cylinders. Under the considered operating conditions, the normal drag and added-mass forces dominate the hydrodynamic response, whereas tangential frictional drag is comparatively small because of the smooth link surfaces and limited axial velocity gradients. Therefore, the Morison equation is adopted to describe the dominant hydrodynamic forces, providing a practical balance between physical representation and modeling complexity for dynamic simulation and controller evaluation.

Let the equivalent length and diameter of the i -th link be $L_{i}$ and $D_{i}$ , respectively. Its cross-sectional area is given by:

$$A_{i}=\frac{\pi D_{i}^{2}}{4}$$

where $L_{i}$ and $D_{i}$ are the equivalent hydrodynamic parameters determined according to the geometric dimensions of the link. Since the surface of the manipulator links is relatively smooth and the fluid velocity gradient along the link axis is small under normal underwater operation speeds, this paper mainly considers the normal fluid force and neglects the influence of tangential friction resistance on the overall hydrodynamic model.

For the i -th link, an infinitesimal element ds is selected at the axial position $s\in \left[0,L_{i}\right]$ . Let the normal velocity and normal acceleration of this element relative to the water be $v_{n,i}\left(s\right)$ and $a_{n,i}\left(s\right)$ , respectively. According to the Morison equation, the hydrodynamic force acting on this infinitesimal element can be expressed as the sum of the drag term and the added-mass term:

$$dF_{h,i}=dF_{D,i}+dF_{M,i}$$

where the infinitesimal drag force is:

$$dF_{D,i}=-\frac{1}{2}\rho C_{D}D_{i}v_{n,i}\left(s\right)|v_{n,i}\left(s\right)|ds$$

and the infinitesimal added-mass force is:

$$dF_{M,i}=-\rho C_{M}A_{i}a_{n,i}\left(s\right)ds$$

where ρ is the water density, $C_{D}$ is the drag coefficient, and $C_{M}$ is the inertia coefficient. The negative signs indicate that the drag force acts opposite to the relative velocity, whereas the added-mass force acts opposite to the relative acceleration. Since the manipulator links are approximated as slender circular cylinders, literature-reported representative values for cylindrical members in Morison-equation-based hydrodynamic analyses were adopted. Accordingly, the seawater density was set to $\rho =1025\text{ kg}/m^{3}$ , while the drag coefficient and Morison inertia coefficient were specified as $C_{d}=1.0$ and $C_{m}=2.0$ , respectively (Vugts et al., 2001).

Therefore, the total hydrodynamic force acting on the link element can be written as:

$$dF_{h,i}=-\left[\frac{1}{2}\rho C_{D}D_{i}v_{n,i}\left(s\right)|v_{n,i}\left(s\right)|+\rho C_{M}A_{i}a_{n,i}\left(s\right)\right]ds$$

This equation indicates that the hydrodynamic force is mainly composed of a nonlinear drag term related to the square of velocity and an added-mass term related to acceleration. The drag term reflects the dissipative effect of water on link motion, while the added-mass term represents the equivalent inertial effect generated when the accelerating link drives the surrounding fluid.

By integrating along the full length of the i -th link, the total hydrodynamic force acting on the link can be obtained as:

$$F_{h,i}=\int_{0}^{L_{i}}dF_{h,i}=-\int_{0}^{L_{i}}\left[\frac{1}{2}\rho C_{D}D_{i}v_{n,i}\left(s\right)|v_{n,i}\left(s\right)|+\rho C_{M}A_{i}a_{n,i}\left(s\right)\right]ds$$

In the dynamic model of the manipulator, the hydrodynamic force needs to be further transformed into the equivalent torque in joint space. Let $r_{ij}\left(s\right)$ denote the equivalent moment arm from the link element to the axis of the j -th joint. The hydrodynamic torque generated by this element on joint j can then be expressed as:

$$d\tau_{h,ij}=r_{ij}\left(s\right)dF_{h,i}$$

By integrating along the full length of the link, the equivalent hydrodynamic torque generated by the i -th link on the j -th joint can be obtained as:

$$\tau_{h,ij}=\int_{0}^{L_{i}}r_{ij}\left(s\right)dF_{h,i}$$

which can be further expanded as:

$$\tau_{h,ij}=-\int_{0}^{L_{i}}r_{ij}\left(s\right)\left[\frac{1}{2}\rho C_{D}D_{i}v_{n,i}\left(s\right)|v_{n,i}\left(s\right)|+\rho C_{M}A_{i}a_{n,i}\left(s\right)\right]ds$$

Therefore, the hydrodynamic torque can be decomposed into the drag-induced torque and the added-mass-induced torque:

$$\tau_{h,ij}=\tau_{D,ij}+\tau_{M,ij}$$

$$\begin{array}{l} \tau_{D,ij}=-\int_{0}^{L_{i}}r_{ij}\left(s\right)\frac{1}{2}\rho C_{D}D_{i}v_{n,i}\left(s\right)|v_{n,i}\left(s\right)|ds\tau_{M,ij}\\ \;=-\int_{0}^{L_{i}}r_{ij}\left(s\right)\rho C_{M}A_{i}a_{n,i}\left(s\right)ds \end{array}$$

For the 6-DOF serial underwater manipulator, the hydrodynamic torques generated by all links jointly act in the joint space. By summing the hydrodynamic torques generated by all links on each joint, the overall generalized hydrodynamic torque can be obtained as:

$$\tau_{h}=\sum_{i=1}^{6}\tau_{h,i}=\tau_{D}+\tau_{M}$$

where $\tau_{D}$ denotes the equivalent joint torque generated by fluid drag, and $\tau_{M}$ denotes the equivalent joint torque generated by the added-mass effect.

In the implemented simulation model, $\tau_{D}$ and $\tau_{M}$ are calculated separately by the underwater-environment module according to the instantaneous motion states of the manipulator and are applied only once as external joint torques. No separate added-mass matrix or hydrodynamic damping matrix is additionally superimposed. Therefore, the added-mass and drag effects are not counted repeatedly.

This generalized hydrodynamic torque can be further introduced into the dynamic model of the underwater manipulator:

$$M_{r}\left(q\right)\ddot{q}+C_{r}\left(q,\dot{q}\right)\dot{q}+G_{b}\left(q\right)=\tau +\tau_{h}$$

where τ is the joint driving torque generated by the controller, and $\tau_{h}$ is the signed external hydrodynamic torque composed of the drag-induced torque and the added-mass-induced torque. The direction of $\tau_{h}$ is determined by the relative velocity and acceleration terms in the Morison equation.

Through the Morison-equation-based underwater-environment module, the fluid drag and added-mass effects are calculated explicitly and introduced once into the rigid-body manipulator model as external joint torques. This formulation provides the hydrodynamic basis for the subsequent trajectory tracking controller design and underwater disturbance simulation.

### 3.4 Ocean-current disturbance model

During underwater operations, the manipulator is affected not only by hydrodynamic forces generated by its own motion, but also by external ocean currents and wave-induced disturbances. Such disturbances usually exhibit persistent and low-frequency variation characteristics, which can easily cause joint trajectory deviations and end-effector tracking errors. To simulate the influence of external disturbances under complex sea conditions, this paper constructs a compound ocean-current disturbance model composed of a constant bias load and a low-frequency surge disturbance:

$$\tau_{c}\left(t\right)=\tau_{s}+A\sin\left(\omega t\right)$$

where $\tau_{c}\left(t\right)$ is the ocean-current disturbance signal, $\tau_{s}$ is the persistent bias load caused by a constant ocean current, A is the amplitude of the low-frequency surge disturbance, and ω is the angular frequency of the disturbance. The constant term is used to describe the persistent external load caused by a steady current, while the sinusoidal term is used to simulate periodic disturbances induced by waves or surges.

Considering that the links, loads, and flow-exposed areas of different joints of the 6-DOF manipulator are different, the equivalent disturbance torques acting on each joint under the same ocean current are not identical. Therefore, the above disturbance signal is mapped into a disturbance torque vector in joint space:

$$\tau_{\text{dist}}\left(t\right)=\Gamma \tau_{c}\left(t\right)=\Gamma \left[\tau_{s}+A\sin\left(\omega t\right)\right]$$

where $\tau_{\text{dist}}\left(t\right)\in R^{6}$ is the ocean-current disturbance torque in joint space, and $\Gamma ={\left[\gamma_{1},\gamma_{2},\ldots ,\gamma_{6}\right]}^{T}$ is the joint disturbance distribution coefficient vector, which describes the relative influence of ocean currents on different joints.

The relative distribution coefficient of the i -th joint is determined based on the hydrodynamic loads acting on its downstream links and the corresponding effective moment arms:

$$\gamma_{i}=\frac{\sum_{j=i}^{6}S_{j}l_{ij}}{\underset{k=1,\ldots ,6}{\max}\left(\sum_{j=k}^{6}S_{j}l_{kj}\right)}$$

where $S_{j}$ denotes the equivalent flow-exposed area of the j -th link, and $l_{ij}$ denotes the effective moment arm from the hydrodynamic force center of the j -th link to the i -th joint. After normalization, $\gamma_{i}$ represents the relative intensity of the ocean-current disturbance acting on each joint. Since the base, shoulder, and elbow joints are subjected to larger cumulative hydrodynamic loads from the downstream links and longer effective moment arms, the distribution coefficients of Joints 1–3 are relatively large. In contrast, the wrist joints have smaller flow-exposed areas and effective moment arms; therefore, the distribution coefficients of Joints 4–6 are relatively small.

In the Simulink model, the constant bias and low-frequency periodic disturbance signals are generated using the Constant and Sine Wave blocks, respectively. After being superimposed through an Add block, the combined disturbance is converted into a 6×1 joint disturbance torque vector through a Gain block according to the above joint-wise distribution relationship. The same disturbance model and distribution method are applied to all control methods to ensure a fair comparison.

After introducing the ocean-current disturbance, the dynamic model of the underwater manipulator can be written as:

$$M_{r}\left(q\right)\ddot{q}+C_{r}\left(q,\dot{q}\right)\dot{q}+G_{b}\left(q\right)=\tau +\tau_{h}+\tau_{\text{dist}}\left(t\right)$$

where $\tau_{h}$ denotes the hydrodynamic torque generated by the underwater motion of the manipulator itself, and $\tau_{\text{dist}}\left(t\right)$ denotes the equivalent disturbance torque caused by external ocean currents.

Through this compound disturbance model, an underwater disturbance environment with persistent bias and low-frequency periodic variation characteristics can be constructed in simulation, which is used to evaluate the trajectory tracking accuracy and disturbance rejection performance of the controller.

## 4 Trajectory tracking control method based on IPSO-PID

### 4.1 Joint trajectory tracking control framework for the underwater manipulator

**FIGURE 2:**
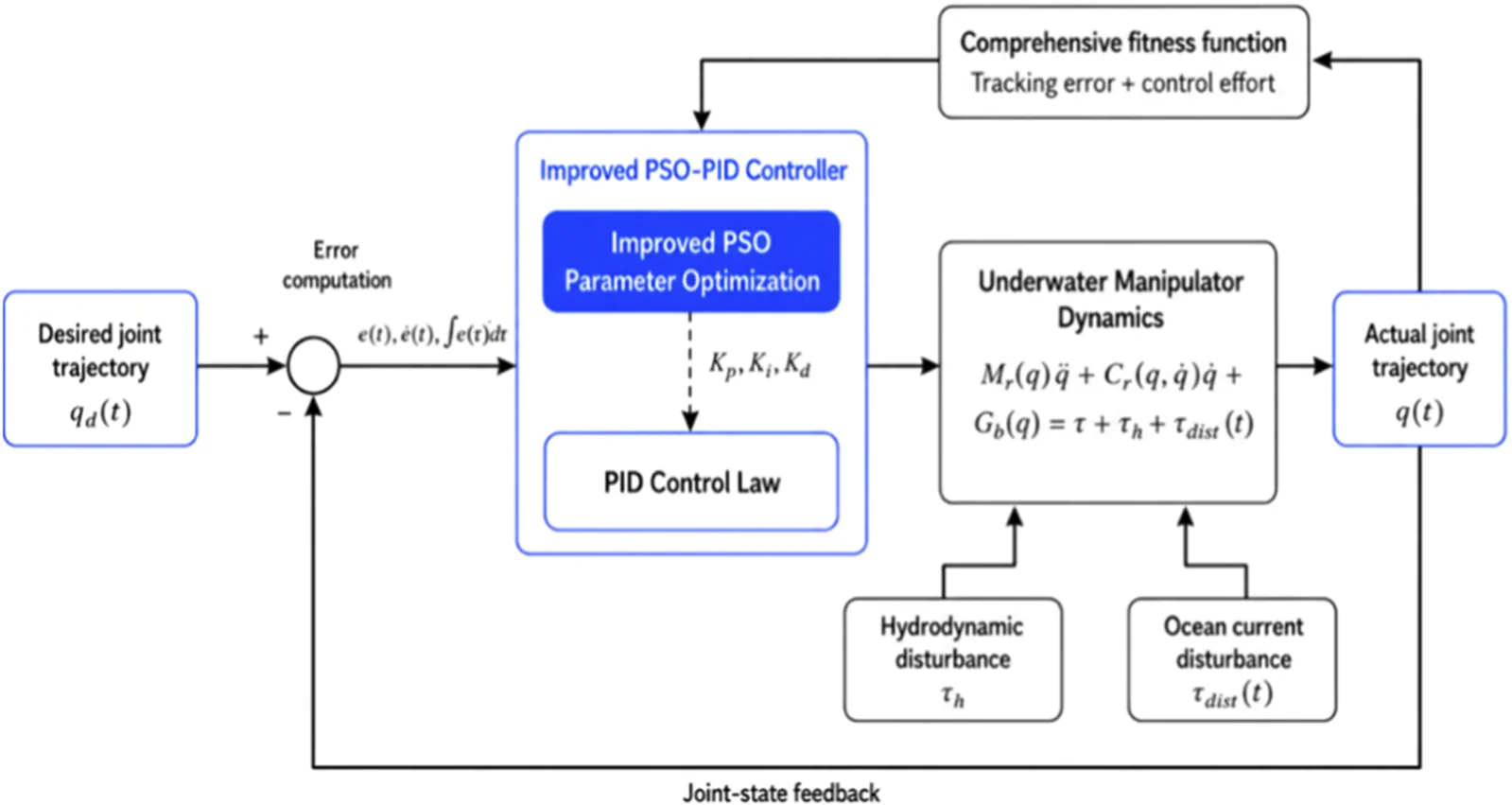
IPSO-PID joint trajectory tracking control framework for the underwater manipulator.

To improve the trajectory tracking performance of the underwater manipulator under hydrodynamic loads and external ocean-current disturbances, this paper constructs a joint trajectory tracking control framework based on IPSO-PID, as shown in Figure 2. The framework takes the desired joint trajectory $q_{d}\left(t\right)$ as the input, obtains the actual joint trajectory $q\left(t\right)$ through joint state feedback, and calculates the trajectory tracking error, error derivative, and error integral. Then, the IPSO-PID controller generates joint control torques according to the optimized PID parameters, thereby driving the underwater manipulator to perform closed-loop trajectory tracking.

The joint trajectory tracking error is defined as:

$$e\left(t\right)=q_{d}\left(t\right)-q\left(t\right)$$

The error derivative is expressed as:

$$\dot{e}\left(t\right)={\dot{q}}_{d}\left(t\right)-\dot{q}\left(t\right)$$

In the basic control layer, the PID control law can be written as:

$$\tau \left(t\right)=K_{p}e\left(t\right)+K_{i}\int_{0}^{t}e\left(\xi \right)d\xi +K_{d}\dot{e}\left(t\right)$$

where $\tau \left(t\right)$ is the joint control torque, and $K_{p}$ , $K_{i}$ , and $K_{d}$ are the proportional, integral, and derivative control parameters, respectively. Since the underwater manipulator is affected by multi-joint coupling, hydrodynamic damping, added mass, and ocean-current disturbances, fixed PID parameters are difficult to maintain desirable response speed, tracking accuracy, and disturbance rejection performance under different operating conditions. Therefore, this paper adopts the improved PSO algorithm to automatically optimize the PID parameters.

The improved PSO module uses the comprehensive fitness function as the evaluation criterion, incorporating both the trajectory tracking error and control energy into the optimization objective, so as to search for better combinations of $K_{p}$ , $K_{i}$ , and $K_{d}$ . The optimized PID controller outputs the joint driving torques and forms a closed-loop control system with the dynamic model of the underwater manipulator. In this model, hydrodynamic disturbances and external ocean-current disturbances are considered, which makes it possible to evaluate the trajectory tracking performance and disturbance rejection capability of the controller in complex underwater environments.

Through the above control framework, the system can automatically optimize PID parameters according to the trajectory tracking performance, thereby improving the trajectory tracking accuracy and stability of the underwater manipulator in complex disturbance environments.

### 4.2 PID parameter optimization and comprehensive fitness function design

For the 6-DOF underwater manipulator, the proportional, integral, and derivative parameters of the PID controller directly affect joint trajectory tracking accuracy, error convergence speed, and the smoothness of control inputs. Since dynamic coupling exists among different joints, independently tuning the parameters of each joint is difficult to achieve globally optimal control performance. Therefore, this paper constructs the PID parameters of all six joints as unified optimization variables and uses the improved PSO algorithm for cooperative optimization.

The PID parameters of the six joints are arranged sequentially according to the proportional, integral, and derivative terms, forming an 18-dimensional optimization vector:

$$X=\left[K_{p1},K_{p2},\ldots ,K_{p6},K_{i1},K_{i2},\ldots ,K_{i6},K_{d1},K_{d2},\ldots ,K_{d6}\right]$$

where $K_{pj}$ , $K_{ij}$ , and $K_{dj}$ denote the proportional, integral, and derivative parameters of the j -th joint, respectively, with j=1,2,…,6 . During the optimization process, the position of each particle corresponds to a complete set of PID parameter combinations for all six joints. To avoid unreasonable control gains, the optimization variables must satisfy the following boundary constraints:

$$X_{\min}\leq X\leq X_{\max}$$

where $X_{\min}$ and $X_{\max}$ are the lower and upper bounds of the PID parameter search range, respectively.

To comprehensively evaluate the control performance under different PID parameter combinations, this paper constructs a fitness function that includes the tracking error, error attenuation speed, and control energy:

$$J=\alpha J_{\text{IAE}}+\beta J_{\text{ITAE}}+\gamma J_{u}$$

where $J_{\text{IAE}}$ is the integral of absolute error, which is used to evaluate the overall trajectory tracking accuracy; $J_{\text{ITAE}}$ is the integral of time-weighted absolute error, which emphasizes the error convergence performance in the later stage; $J_{u}$ is the control energy term, which is used to limit excessive joint control inputs; The weighting coefficients of the fitness function were set to α=1.0 , β=0.5 , and $\gamma =5\times 10^{-4}$ , respectively.

Specifically, these performance indices are defined as:

$$J_{\text{IAE}}=\sum_{j=1}^{6}w_{j}\int_{0}^{T}|e_{j}\left(t\right)|dt$$

$$J_{\text{ITAE}}=\sum_{j=1}^{6}w_{j}\int_{j=1}^{6}t|e_{j}\left(t\right)|dt$$

$$J_{u}=\sum_{j=1}^{6}\int_{0}^{T}\tau_{j}^{2}\left(t\right)dt$$

where $e_{j}\left(t\right)$ denotes the trajectory tracking error of the j -th joint, $\tau_{j}\left(t\right)$ denotes the control torque of the j -th joint, $w_{j}$ is the joint error weighting coefficient, and T is the simulation time. A smaller fitness value indicates that the corresponding PID parameter set achieves better overall performance in terms of trajectory tracking accuracy, error attenuation, and control energy consumption.

Therefore, the PID parameter optimization problem can be formulated as:

$$\begin{array}{l} X^{*}=\arg\underset{X}{\min}J\left(X\right)\\ s.t.X_{\min}\leq X\leq X_{\max} \end{array}$$

where $X^{*}$ denotes the optimal PID parameter combination. Through this optimization model, PID parameter design is transformed from manual empirical tuning into a constrained multivariable optimization problem, providing the objective function and evaluation criterion for the subsequent parameter search using the improved PSO algorithm.

### 4.3 Improved particle swarm optimization algorithm

Particle swarm optimization searches for the optimal solution in the parameter space by simulating the cooperative search mechanism of a swarm. In PID parameter optimization, each particle represents a candidate set of PID parameters. The particle position corresponds to the controller parameter combination, and the fitness value is used to evaluate the trajectory tracking performance obtained with that parameter set. In standard PSO, the position and velocity of the i -th particle at the k -th iteration are denoted as $X_{i}^{k}$ and $V_{i}^{k}$ , respectively. The update process can be expressed as:

$$\begin{array}{l} V_{i}^{k+1}=\omega V_{i}^{k}+c_{1}r_{1}\left(P_{i}^{k}-X_{i}^{k}\right)+c_{2}r_{2}\left(G^{k}-X_{i}^{k}\right)\\ \;X_{i}^{k+1}=X_{i}^{k}+V_{i}^{k+1} \end{array}$$

where ω is the inertia weight, $c_{1}$ and $c_{2}$ are the individual and social learning factors, respectively, $r_{1}$ and $r_{2}$ are random numbers within $\left[0,1\right]$ , $P_{i}^{k}$ is the personal best position of the i -th particle, and $G^{k}$ is the global best position of the particle swarm.

In standard PSO, w , $c_{1}$ , and $c_{2}$ are usually fixed constants. For the high-dimensional PID parameter optimization problem of a six-joint underwater manipulator, fixed parameter settings may cause particles to aggregate prematurely, resulting in reduced population diversity and insufficient local search capability. Therefore, the parameter adjustment strategy is designed according to different optimization stages. In the early stage, a larger inertia weight and stronger individual learning capability are used to expand the search range and reduce the risk of premature convergence. In the later stage, the inertia weight is gradually decreased and the social learning capability is strengthened to guide the particles toward the global optimum and improve local search accuracy. Based on this design logic, a linearly decreasing inertia weight and adaptive learning factors are introduced.

First, a linearly decreasing inertia weight is adopted to adjust the search step size of the particles:

$$\omega \left(k\right)=\omega_{\max}-\frac{\omega_{\max}-\omega_{\min}}{\text{MaxIt}-1}\left(k-1\right)$$

where $\omega_{\max}$ and $\omega_{\min}$ are the maximum and minimum values of the inertia weight, respectively, MaxIt is the maximum number of iterations, and k is the current iteration number. This strategy enables the particles to maintain a larger search step size in the early optimization stage, thereby enhancing global exploration. In the later optimization stage, the inertia weight gradually decreases, allowing the particles to perform more refined local search in promising regions.

Second, asymmetric adaptive learning factors are adopted. The individual learning factor $c_{1}$ gradually decreases with the iteration process, while the social learning factor $c_{2}$ gradually increases:

$$c_{1}\left(k\right)=c_{1\;\max}-\frac{c_{1\max}-c_{1\min}}{\text{MaxIt}-1}\left(k-1\right)$$

$$c_{2}\left(k\right)=c_{2\min}+\frac{c_{2\max}-c_{2\min}}{\text{MaxIt}-1}\left(k-1\right)$$

where $c_{1\max}$ , $c_{1\min}$ , $c_{2\max}$ , and $c_{2\min}$ are the upper and lower limits of the learning factors. In the early iterations, a larger $c_{1}$ makes the particles rely more on their own search experience, which helps maintain population diversity. Meanwhile, a smaller $c_{2}$ prevents the particles from converging prematurely toward the global best position. As the iteration proceeds, $c_{1}$ gradually decreases and $c_{2}$ gradually increases, so that the swarm increasingly exploits the global optimal information, thereby improving convergence stability.

Accordingly, the improved particle velocity update equation can be written as:

$$\begin{array}{l} V_{i}^{k+1}=\omega \left(k\right)V_{i}^{k}+c_{1}\left(k\right)r_{1}\left(P_{i}^{k}-X_{i}^{k}\right)+c_{2}\left(k\right)r_{2}\left(G^{k}-X_{i}^{k}\right)\\ \;X_{i}^{k+1}=X_{i}^{k}+V_{i}^{k+1} \end{array}$$

In each iteration, boundary checking is performed after the particle position is updated. If a parameter exceeds the preset range, it is constrained within the allowable search interval to avoid unreasonable control gains. The updated PID parameters are then input into the underwater manipulator trajectory tracking simulation model, and the fitness value of the current particle is calculated according to the comprehensive fitness function. By continuously updating the personal best position and the global best position, the optimal PID parameter combination suitable for trajectory tracking control of the 6-DOF underwater manipulator is finally obtained.

The proposed improvement strategy enhances the exploration capability of the parameter space in the early optimization stage and improves the local convergence accuracy in the later stage. Therefore, it can alleviate the insufficient search capability and parameter aggregation problems of standard PSO in high-dimensional PID parameter optimization. The obtained PID parameters are then used for the subsequent design of the IPSO-PID controller.

### 4.4 Design of the IPSO-PID controller

**FIGURE 3:**
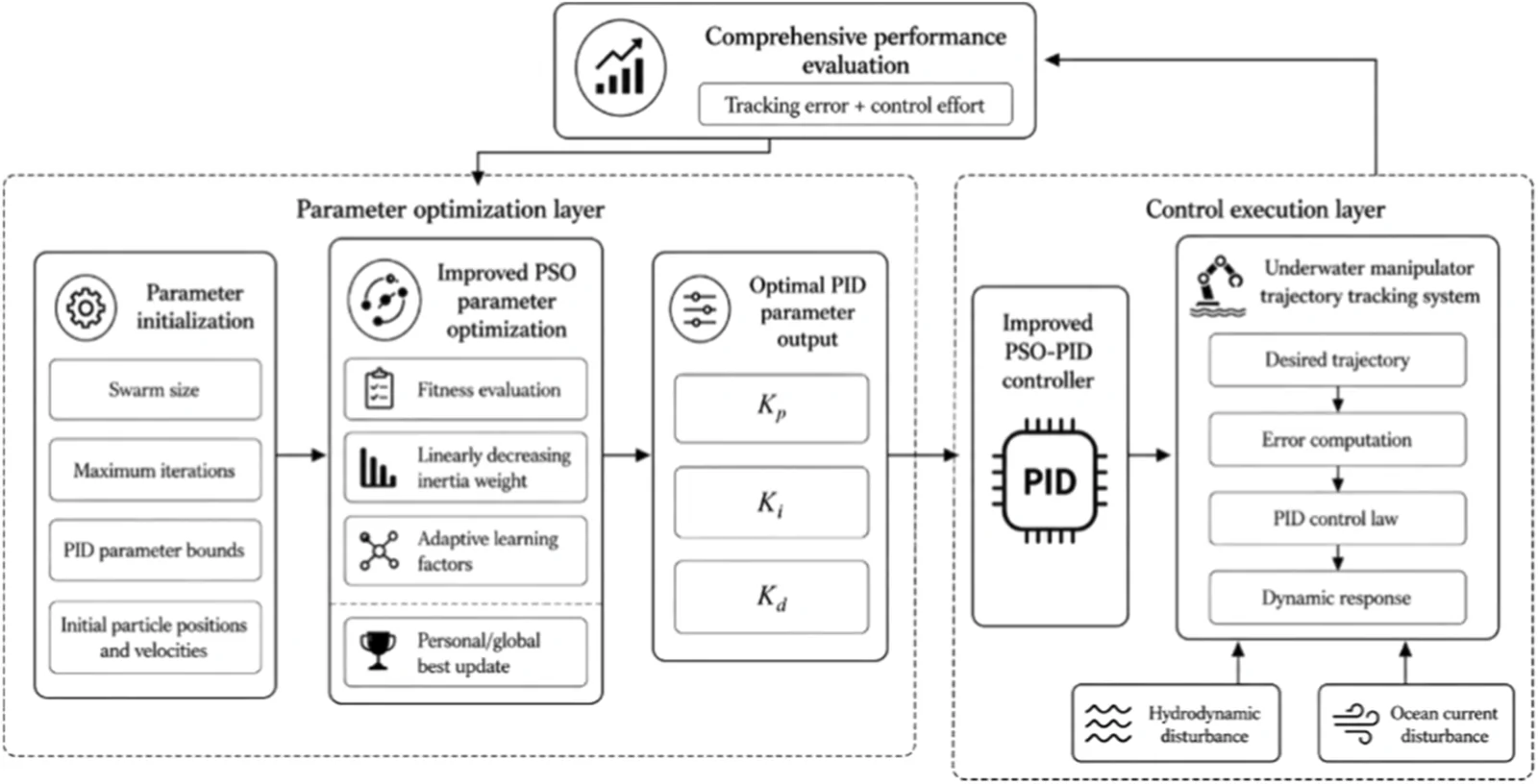
Design flow of the IPSO-PID controller.

After completing the PID parameter optimization model and the design of the improved particle swarm optimization algorithm, this paper further constructs the IPSO-PID controller. The overall implementation process is shown in Figure 3. The controller mainly consists of a parameter optimization layer and a control execution layer. In the parameter optimization layer, the particle swarm size, maximum number of iterations, PID parameter search boundaries, and initial particle positions and velocities are first initialized. Then, each candidate set of PID parameters is input into the MATLAB/Simulink trajectory tracking model, and its control performance is evaluated according to the comprehensive fitness function. Finally, the improved PSO particle update mechanism is used to iteratively search for better PID parameter combinations, and the optimal $K_{p}$ , $K_{i}$ , and $K_{d}$ values are obtained.

Algorithm 1 IPSO-PID parameter optimization procedure.

**Input:** D=18 , N=25 , MaxIt=20 , PID parameter bounds
**Output:** Optimal PID parameter vector $X_{\text{best}}$

1: Initialize particle positions and velocities in the D -dimensional parameter space.
2: Evaluate the initial particles and initialize $P_{\text{best}}$ and $G_{\text{best}}$ .
3: **for** k=1 to MaxIt **do**
4: Update the inertia weight and adaptive learning factors.
5: Update particle velocities and positions and apply boundary constraints.
6: Run the MATLAB/Simulink model and calculate the fitness values.
7: Update $P_{\text{best}}$ and $G_{\text{best}}$ .
8: **end for**
9: Return $G_{\text{best}}$ as $X_{\text{best}}$ .

In the control execution layer, the optimized PID parameters are loaded into the joint trajectory tracking controller to generate the joint control torques of the underwater manipulator. The actual joint response is output through the dynamic model of the underwater manipulator and then fed back to the error calculation module, forming a closed-loop control system. This design combines offline parameter optimization with closed-loop trajectory tracking control. It maintains the simple structure of PID control while improving the trajectory tracking accuracy and robustness of the underwater manipulator under hydrodynamic and ocean-current disturbances.

## 5 Simulation platform and experimental setup

### 5.1 MATLAB/Simulink simulation platform for the underwater manipulator

**FIGURE 4:**
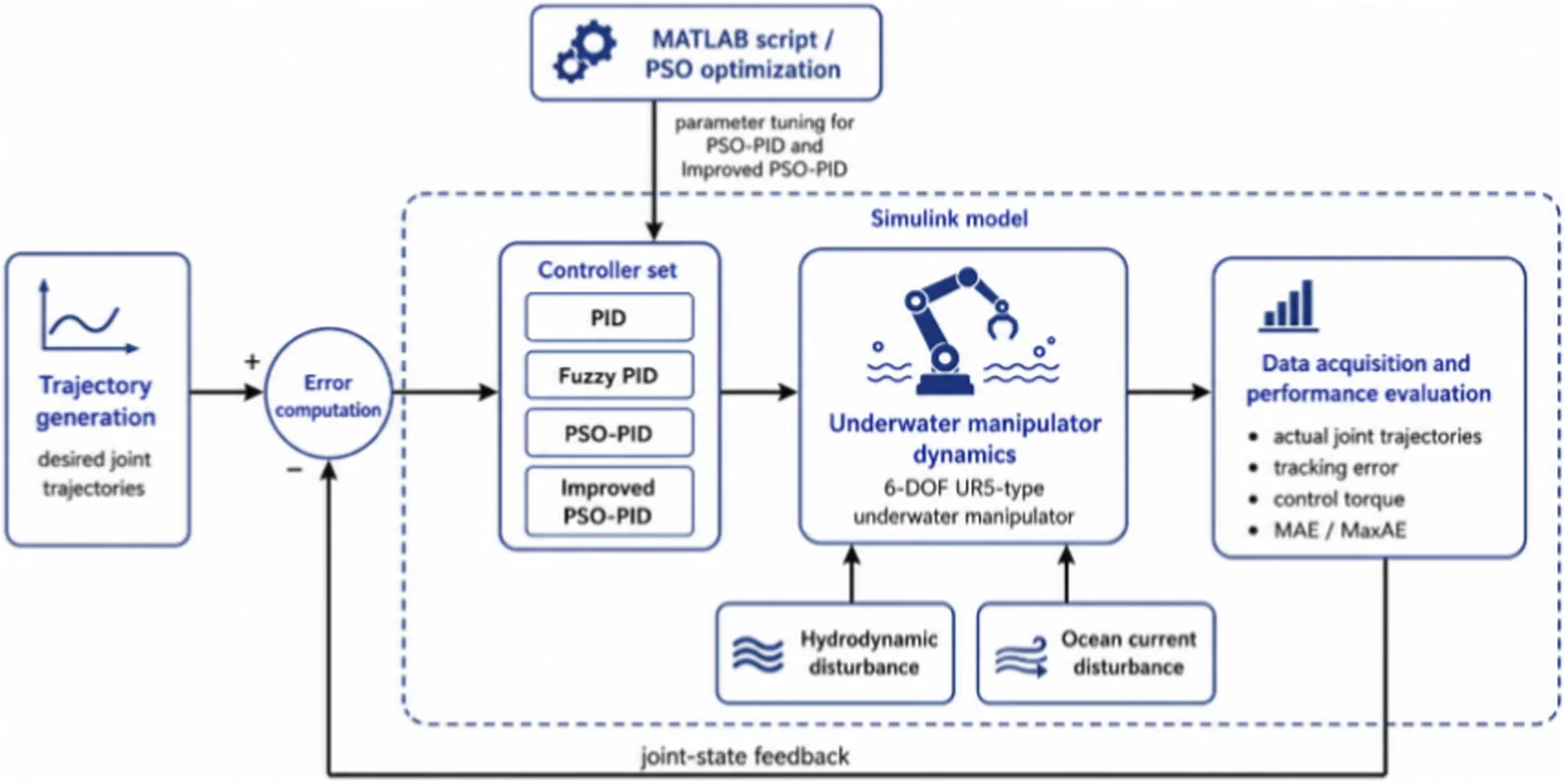
MATLAB simulation platform for underwater manipulator trajectory tracking.

To verify the effectiveness of the proposed IPSO-PID control method in the trajectory tracking task of the underwater manipulator, a simulation platform for a 6-DOF underwater manipulator is established based on MATLAB/Simulink. Its overall structure is shown in Figure 4. The platform takes a UR5-type 6-DOF underwater manipulator as the controlled object and mainly includes the trajectory generation module, error calculation module, controller module, underwater manipulator dynamic model, environmental disturbance input, and data acquisition and performance evaluation module. Through this platform, PID, fuzzy PID, PSO-PID, and IPSO-PID control methods can be compared and analyzed under the same model and simulation conditions.

During the simulation, the trajectory generation module provides the reference trajectories for the six joints, and the error calculation module obtains the error signals required for control input according to the desired trajectories and actual joint responses. The parameter optimization of PSO-PID and IPSO-PID is jointly completed using MATLAB scripts and the Simulink model. Specifically, the MATLAB scripts are responsible for particle swarm iteration and parameter updating, while the Simulink model is used to calculate the joint responses and tracking errors under the current PID parameters. The manipulator model represents the rigid-body dynamics and the gravity–buoyancy effect. The underwater-environment module separately calculates the Morison-based drag and added-mass torques and applies them once as external joint torques. The ocean-current disturbance is introduced through the environmental disturbance module. This provides the simulation basis for subsequent analysis of trajectory tracking accuracy and disturbance rejection performance.

### 5.2 Desired trajectories and control parameter settings

To ensure consistency in the comparison among different control methods, trajectory tracking experiments are conducted under the same underwater manipulator model, reference trajectories, and simulation time. The desired trajectories of the six joints are generated using sine signal modules in MATLAB/Simulink, which are used to simulate the trajectory tracking process of the underwater manipulator during periodic joint motion. The simulation time is set to 20 s, and four control methods, namely PID, fuzzy PID, PSO-PID, and IPSO-PID, are compared.

The desired trajectory of the j -th joint is defined as:

$$q_{d,j}\left(t\right)=A_{j}\sin\left(\omega_{j}t+\phi_{j}\right)+q_{0,j}$$

where $A_{j}$ is the motion amplitude of the j -th joint, $\omega_{j}$ is the angular frequency, $\phi_{j}$ is the initial phase, and $q_{0,j}$ is the joint angle offset, with j=1,2,…,6 . By assigning different trajectory parameters to different joints, a trajectory tracking task under multi-joint synchronous motion can be constructed, thereby evaluating the tracking capability of the controller for periodic reference inputs.

For both PSO-PID and IPSO-PID, each particle is represented by an 18-dimensional vector containing the proportional, integral, and derivative gains of all six joints. The population size and maximum number of iterations are set to 25 and 20, respectively. The joint error weights used in the IAE and ITAE terms are set to $w=\left[1.0,1.4,1.4,1.2,0.8,0.8\right]$ , corresponding to Joints 1–6, respectively. The same particle dimension, population size, iteration number, joint error weights, and PID parameter search boundaries are used for both optimization algorithms to ensure a fair comparison.

### 5.3 Comparative control method settings

To systematically evaluate the trajectory tracking performance of the proposed IPSO-PID control method, four control methods are compared in this paper, including PID, fuzzy PID, PSO-PID, and IPSO-PID. All four methods are tested using the same underwater manipulator dynamic model, reference trajectories, and simulation time to ensure the fairness of the comparison.

The conventional PID controller is used as the baseline method. Its parameters are mainly determined based on manual tuning results, so as to reflect the basic performance of fixed-parameter control in the trajectory tracking task of the underwater manipulator. The fuzzy PID controller introduces fuzzy rules on the basis of conventional PID control and adjusts the control parameters according to the trajectory error and error change rate, thereby improving the adaptability of the controller to nonlinear dynamic variations. The standard PSO-PID controller uses the particle swarm optimization algorithm to automatically optimize PID parameters, reducing the uncertainty caused by manual parameter tuning. The IPSO-PID controller further introduces a linearly decreasing inertia weight and adaptive learning factors into the standard PSO framework to enhance parameter search capability and convergence stability.

The performance of different control methods is mainly evaluated using joint trajectory tracking errors. In this paper, the maximum absolute error and mean absolute error are adopted as the main quantitative indicators. The maximum absolute error is used to reflect the peak deviation during trajectory tracking, while the mean absolute error is used to evaluate the overall tracking accuracy over the entire simulation period. In the subsequent result analysis, the error reduction ratio is further used to evaluate the performance improvement of the IPSO-PID method compared with PID, fuzzy PID, and standard PSO-PID.

## 6 Simulation results and analysis

### 6.1 Comparison of trajectory tracking results between PID and fuzzy PID

To preliminarily analyze the influence of different control strategies on the joint trajectory tracking performance of the underwater manipulator, this paper first compares the conventional PID controller with the fuzzy PID controller. Both control methods are tested on the same MATLAB/Simulink simulation platform, under the same six-joint reference trajectories and the same simulation time, to ensure the comparability of the experimental results.

Under conventional PID control, all six joints of the underwater manipulator can basically track the desired trajectories, indicating that the established joint trajectory tracking control system operates properly. However, since the PID controller uses fixed control parameters, its adaptability to nonlinear factors such as multi-joint coupling, hydrodynamic damping, and added mass is limited. Therefore, certain error fluctuations still exist during the trajectory tracking process of some joints, especially for the second and third joints, where the error amplitudes are relatively large.

**FIGURE 5:**
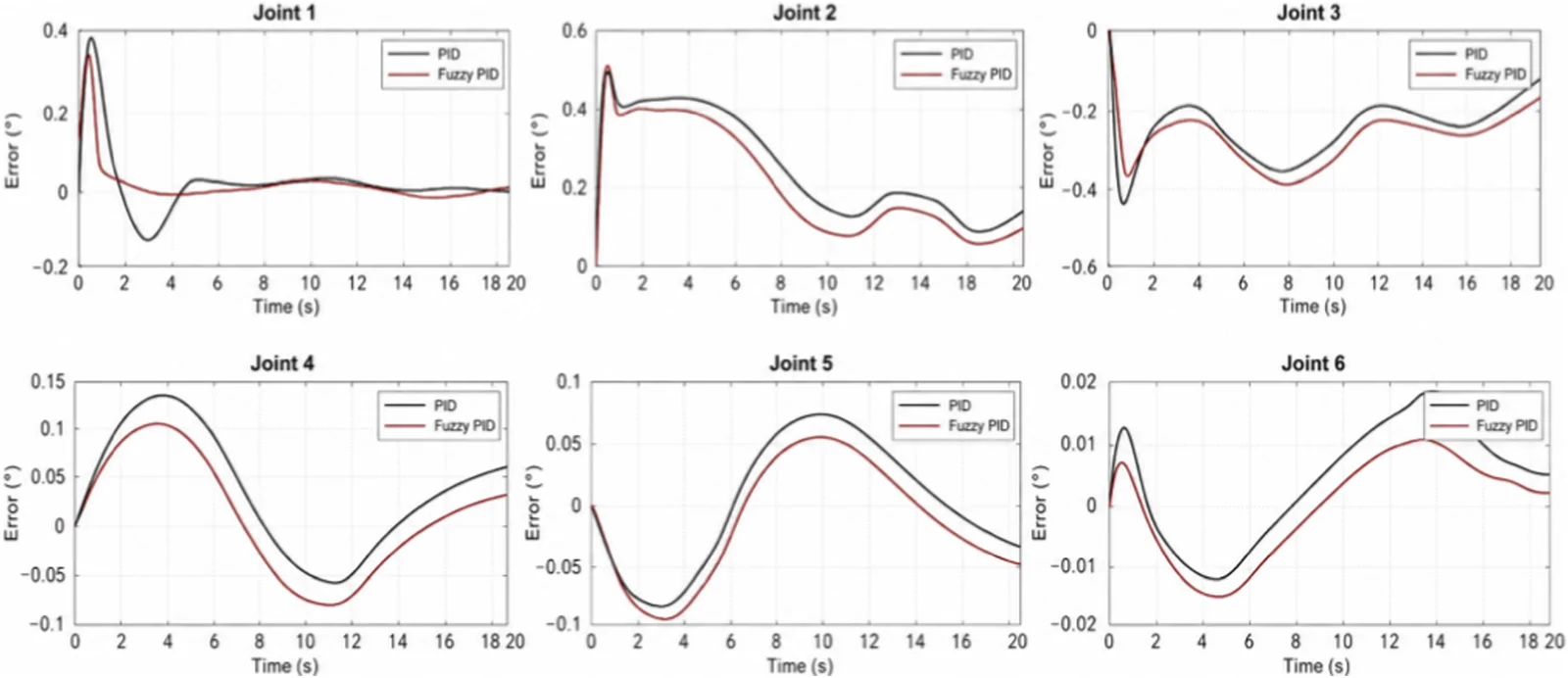
Joint tracking error comparison between PID and fuzzy PID controllers.

As shown in Figure 5, the tracking errors of all six joints under PID control gradually converge, but the error amplitudes vary significantly among different joints. In particular, the second and third joints exhibit relatively larger errors, indicating that conventional PID control has limited error suppression capability for some joints under multi-joint coupling and hydrodynamic effects. In contrast, the fuzzy PID controller introduces fuzzy rules to adjust PID parameters online according to the trajectory error and error change rate, which suppresses the error fluctuations of each joint to some extent. Therefore, its overall tracking performance is better than that of the conventional PID controller.

The quantitative results of the joint trajectory tracking errors of the two control methods are shown in Table 2.

**TABLE 2:** Joint trajectory tracking errors of the PID and fuzzy PID controllers.

Joint	1	2	3	4	5	6
PID maximum absolute error (rad)	0.0069	0.0099	0.0090	0.0026	0.0070	0.0054
PID mean absolute error (rad)	0.0008	0.0041	0.0039	0.0011	0.0023	0.0021
Fuzzy PID maximum absolute error (rad)	0.0066	0.0090	0.0083	0.0021	0.0016	0.0005
Fuzzy PID mean absolute error (rad)	0.0004	0.0033	0.0034	0.0009	0.0007	0.0001

As shown in the joint trajectory tracking error table, under PID control, the maximum joint angle error of the manipulator is 0.0099 rad, and the mean absolute error is 0.0023 rad. Compared with conventional PID, the overall mean absolute error of the fuzzy PID controller is reduced from 0.0023 rad to 0.0014 rad, corresponding to an error reduction of approximately 39.1%. This indicates that the online correction of PID parameters using fuzzy rules can improve the tracking performance of fixed-parameter PID control to a certain extent. However, the control performance of fuzzy PID still depends on the design of fuzzy rules and membership functions. Therefore, PSO-based optimization methods are further adopted in the following section to realize automatic optimization of PID parameters.

### 6.2 Comparison of optimization results between standard PSO-PID and IPSO-PID

To further improve the joint trajectory tracking performance of the underwater manipulator, particle swarm optimization is introduced on the basis of conventional PID parameter tuning. The standard PSO-PID and IPSO-PID methods are then compared and analyzed. Both methods are tested using the same underwater manipulator dynamic model, reference trajectories, and simulation time. The main difference between them lies in the PID parameter optimization strategy. The standard PSO-PID controller uses the conventional particle swarm optimization algorithm for parameter tuning, whereas the IPSO-PID controller introduces a linearly decreasing inertia weight and adaptive learning factors into the standard PSO framework to enhance global search capability and local convergence performance in the later optimization stage.

**FIGURE 6:**
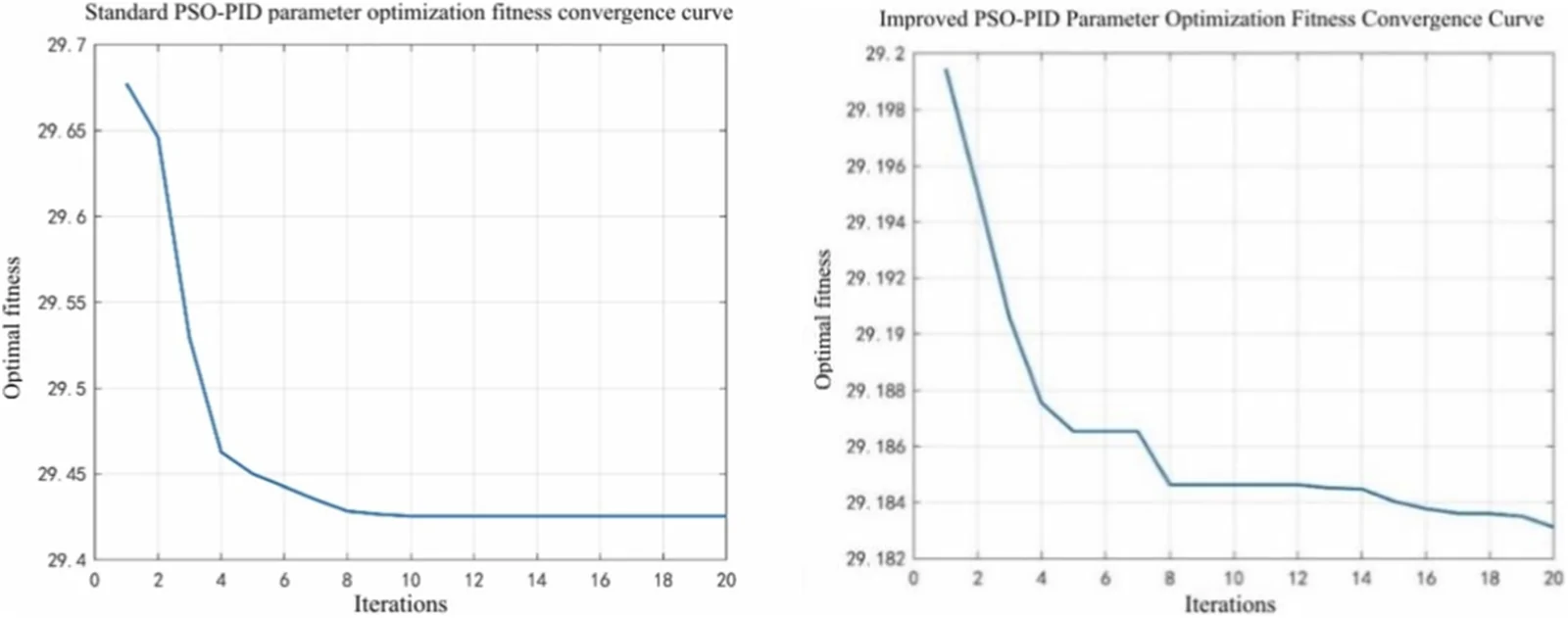
Fitness convergence curves of PSO-PID and IPSO-PID parameter optimization.

As shown in Figure 6, both standard PSO and improved PSO continuously reduce the fitness value during the iteration process, indicating that both methods can effectively optimize the PID parameters. The fitness value of standard PSO decreases rapidly in the first few iterations and becomes stable at approximately the 8th iteration, finally converging to about 29.43. In contrast, the improved PSO also shows a rapid decrease in the early optimization stage, but continues to decrease gradually in the subsequent iterations and finally converges to a lower fitness value of about 29.183. This result indicates that the improved strategy can further enhance the quality of parameter optimization while maintaining search efficiency, thereby obtaining a better PID parameter combination.

**FIGURE 7:**
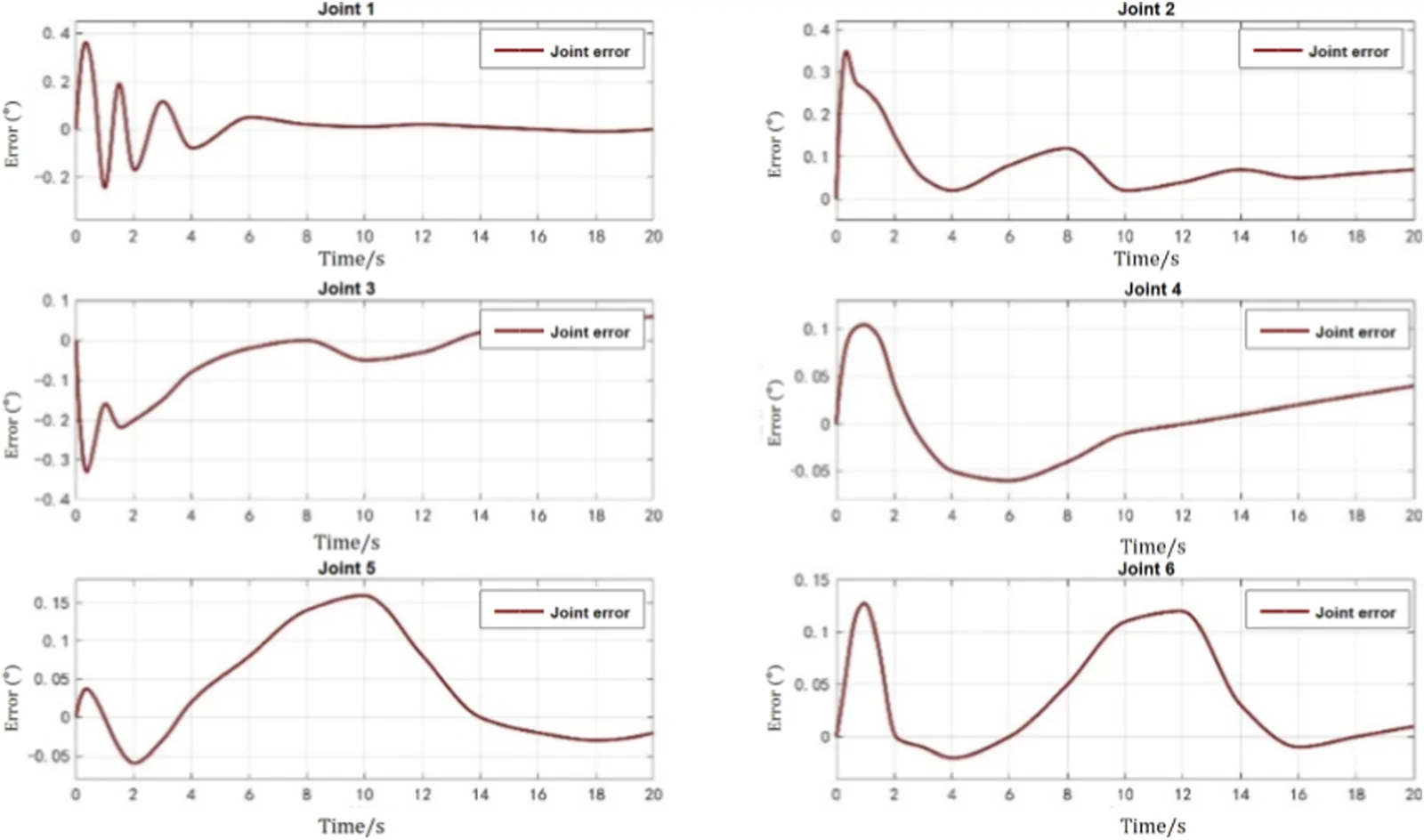
Joint tracking error curves under the standard PSO-PID controller.

**FIGURE 8:**
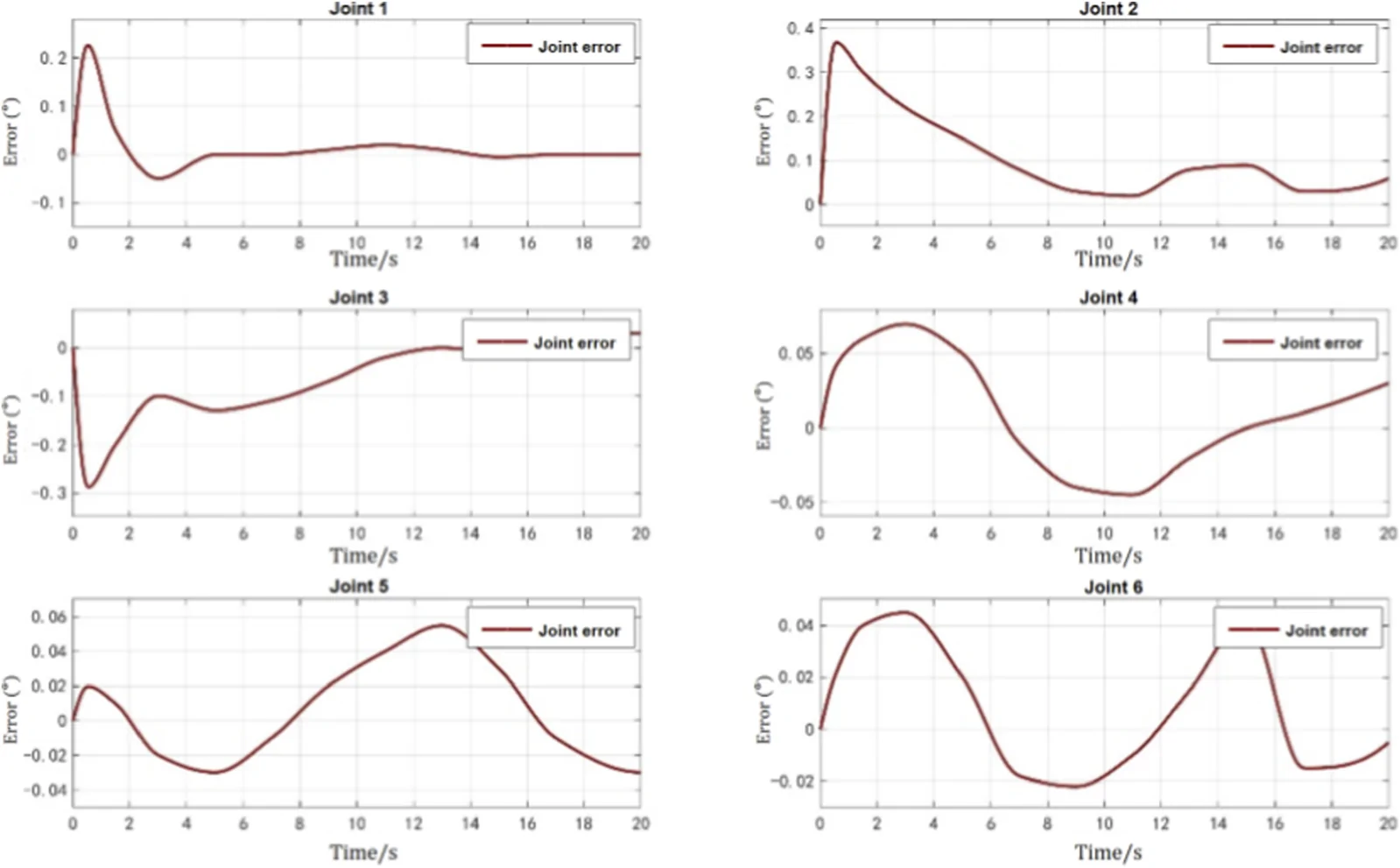
Joint tracking error curves under the IPSO-PID controller.

After the optimized parameters are obtained, the optimal PID parameters generated by standard PSO and improved PSO are applied to the underwater manipulator trajectory tracking control system. The tracking error curves of the six joints are shown in Figures 7, 8, respectively.

The quantitative error results of the two control methods are shown in Table 3.

**TABLE 3:** Joint trajectory tracking errors of the PSO-PID and IPSO-PID controllers.

Joint	1	2	3	4	5	6
PSO-PID maximum absolute error (rad)	0.0066	0.0061	0.0062	0.0013	0.0029	0.0022
PSO-PID mean absolute error (rad)	0.0014	0.0009	0.0016	0.0006	0.0011	0.0007
IPSO-PID maximum absolute error (rad)	0.0038	0.0061	0.0052	0.0011	0.0010	0.0007
IPSO-PID mean absolute error (rad)	0.0007	0.0013	0.0020	0.0005	0.0004	0.0003

To comprehensively evaluate the overall trajectory-tracking performance of the six joints, the overall mean absolute error is defined as the arithmetic mean of the joint-wise mean absolute errors:

$${\text{MAE}}_{\text{overall}}=\frac{1}{6}\sum_{i=1}^{6}{\text{MAE}}_{i}$$

where ${\text{MAE}}_{i}$ denotes the mean absolute error of the i -th joint over all sampling instants.

As shown in Table 3, the maximum absolute error of the standard PSO-PID controller is 0.0066 rad, and its overall mean absolute error is approximately 0.0011 rad. By comparison, the IPSO-PID controller further reduces the maximum absolute error to 0.0061 rad, while the overall mean absolute error is reduced to approximately 0.0008 rad. Compared with standard PSO-PID, the IPSO-PID controller achieves a lower overall mean tracking error, indicating that the improved particle swarm optimization algorithm can enhance the PID parameter optimization effect and improve the comprehensive tracking performance of the controller.

### 6.3 Robustness analysis under compound ocean-current disturbance

**FIGURE 9:**
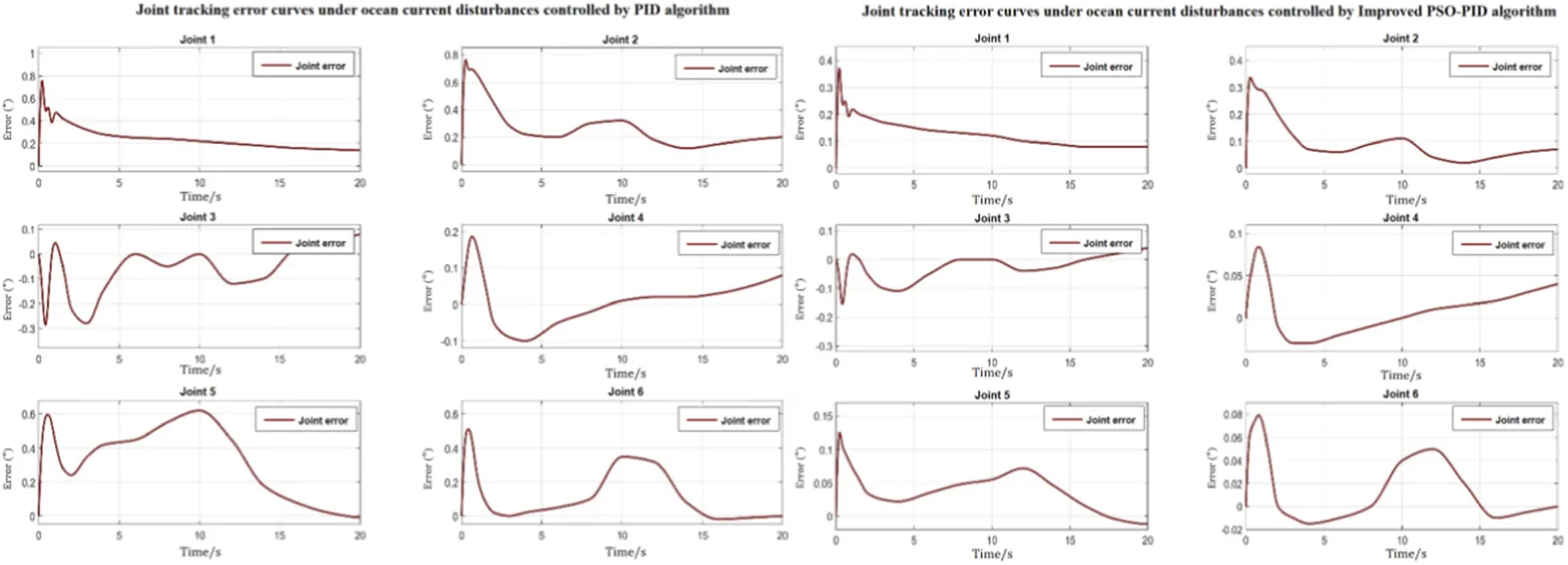
Joint tracking error comparison under compound ocean-current disturbance.

To further verify the disturbance rejection performance of the proposed IPSO-PID control method in complex underwater environments, a compound ocean-current disturbance composed of a constant bias load and a low-frequency periodic disturbance is introduced into the dynamic model of the underwater manipulator. Comparative simulations are then conducted between the conventional PID controller and the IPSO-PID controller. Both methods are tested under the same reference trajectories, simulation time, and disturbance conditions. The joint tracking error results are shown in Figure 9.

As shown in Figure 8, under the compound ocean-current disturbance, the joint errors of the conventional PID controller increase significantly, and low-frequency fluctuations and residual deviations appear in some joints. This indicates that fixed-parameter PID control has limited capability in suppressing persistent bias loads and periodic disturbances. In contrast, the IPSO-PID controller can significantly reduce the error amplitudes of all joints and produce smoother error responses, demonstrating better disturbance rejection capability.

The quantitative results under compound ocean-current disturbance are presented in Table 4. The maximum absolute error of the conventional PID controller reaches 0.0150 rad, and the overall mean absolute error is approximately 0.0044 rad. After adopting the IPSO-PID controller, the maximum absolute error is reduced to 0.0069 rad, while the overall mean absolute error decreases to approximately 0.0014 rad, corresponding to an overall MAE reduction of about 68.2% across all six joints. In particular, the error reductions of Joints 1, 2, 5, and 6 are more pronounced. This improvement is mainly attributed to the more balanced PID gain combination obtained by IPSO, which enhances the correction of disturbance-induced deviations, compensates for the residual error caused by the constant current bias, and suppresses periodic error fluctuations.

**TABLE 4:** Joint trajectory tracking errors under compound ocean-current disturbance.

Joint	1	2	3	4	5	6
PID maximum absolute error (rad)	0.0150	0.0149	0.0057	0.0033	0.0107	0.0122
PID mean absolute error (rad)	0.0057	0.0068	0.0021	0.0014	0.0070	0.0038
IPSO-PID maximum absolute error (rad)	0.0069	0.0061	0.0026	0.0013	0.0022	0.0013
IPSO-PID mean absolute error (rad)	0.0026	0.0027	0.0007	0.0006	0.0009	0.0005

Overall, the compound ocean-current disturbance significantly degrades the trajectory tracking accuracy of the conventional PID controller, whereas the IPSO-PID controller effectively suppresses the increase in joint errors caused by the disturbance. These results further verify the robustness and applicability of the proposed control method in complex underwater environments.

## 7 Conclusion

This paper proposed an improved PSO-optimized PID trajectory tracking control method to address the degradation of tracking accuracy of underwater manipulators under hydrodynamic disturbances and compound ocean-current effects. A 6-DOF underwater manipulator model, including kinematics, hydrodynamics, and ocean-current disturbances, was established, and comparative simulations were conducted on the MATLAB/Simulink platform to validate the effectiveness of the proposed method. The results show that, compared with conventional PID, fuzzy PID, and standard PSO-PID controllers, the IPSO-PID controller further reduces joint trajectory tracking errors and enhances the response stability of the system. Under compound ocean-current disturbance, the overall mean absolute error across all six joints is reduced from approximately 0.0044 rad with conventional PID to approximately 0.0014 rad with the IPSO-PID controller, corresponding to an overall MAE reduction of about 68.2%. Therefore, the proposed method exhibits favorable tracking accuracy and disturbance rejection performance in underwater manipulator trajectory tracking control, and can provide a reference for motion control in underwater robotic grasping, maintenance, and complex marine operations.

The present study is primarily validated through simulations. Future work will establish an experimental platform consisting of a 6-DOF waterproof manipulator, an adjustable-flow recirculating water tank, joint encoders, a six-axis force/torque sensor, and flow-velocity measurement devices. Experiments under still-water, steady-current, periodic-current, and compound-disturbance conditions will be conducted to identify the hydrodynamic parameters and further evaluate the practical tracking accuracy and disturbance-rejection capability of the IPSO-PID controller.

## Data Availability

The raw data supporting the conclusions of this article will be made available by the authors, without undue reservation.
